# Potency of a vaccine prepared from A/swine/Hokkaido/2/1981 (H1N1) against A/Narita/1/2009 (H1N1) pandemic influenza virus strain

**DOI:** 10.1186/1743-422X-10-47

**Published:** 2013-02-06

**Authors:** Masatoshi Okamatsu, Yoshihiro Sakoda, Takahiro Hiono, Naoki Yamamoto, Hiroshi Kida

**Affiliations:** 1 Laboratory of Microbiology, Department of Disease Control, Graduate School of Veterinary Medicine, Hokkaido University, Kita 18 Nishi 9, Kita-ku, Sapporo 060-0818, Japan; 2 Research Center of Zoonosis Control, Hokkaido University, Sapporo, 001-0020, Japan

**Keywords:** Influenza A (H1N1)pdm, Vaccine, Swine influenza virus

## Abstract

**Background:**

The pandemic 2009 (H1N1) influenza virus has spread throughout the world and is now causing seasonal influenza. To prepare for the emergence of pandemic influenza, we have established a library of virus strains isolated from birds, pigs, and humans in global surveillance studies.

**Methods:**

Inactivated whole virus particle (WV) and ether-split (ES) vaccines were prepared from an influenza virus strain, A/swine/Hokkaido/2/1981 (H1N1), from the library and from A/Narita/1/2009 (H1N1) pandemic strain. Each of the vaccines was injected subcutaneously into mice and their potencies were evaluated by challenge with A/Narita/1/2009 (H1N1) virus strain in mice.

**Results:**

A/swine/Hokkaido/2/81 (H1N1), which was isolated from the lung of a diseased piglet, was selected on the basis of their antigenicity and growth capacity in embryonated chicken eggs. Two injections of the WV vaccine induced an immune response in mice, decreasing the impact of disease caused by the challenge with A/Narita/1/2009 (H1N1), as did the vaccine prepared from the homologous strain.

**Conclusion:**

The WV vaccine prepared from an influenza virus in the library is useful as an emergency vaccine in the early phase of pandemic influenza.

## Background

A pandemic influenza caused by swine-origin H1N1 virus appeared in Mexico in 2009 and spread throughout the world [1-3]. The pandemic virus isolates were antigenically similar to classical swine influenza viruses and distinct from H1N1 virus strains circulating in humans since 1977 [2,4]. A pandemic 2009 (H1N1) vaccine was produced and evaluated in clinical trials [5]. The production of a large amount of egg-produced pandemic 2009 (H1N1) vaccine was, however, limited due to its poor yield in chicken embryos [6], leading to a delay in the efficient control of the pandemic.

It was revealed that the H3 HA gene of A/Hong Kong/68 (H3N2) strain originated from that of isolates from migratory ducks and that pigs served as a mixing vessel for the generation of reassortants with the precedent human H2N2 influenza virus [7-10]. To prepare for pandemic influenza, we have conducted a global surveillance of influenza in birds and mammals since 1977, and have established a vaccine strain library of influenza A viruses [11-15]. Their pathogenicity, antigenicity, genetic information, and yield in chicken embryos have been analyzed and the data are available at http://virusdb.czc.hokudai.ac.jp/.

In the present study, a vaccine strain against pandemic 2009 (H1N1) influenza was selected from 42 H1N1 influenza viruses in the virus library. The potency of inactivated whole virus particle (WV) and ether-split (ES) vaccines prepared from a virus strain in the library was evaluated.

## Results

### Antigenic analysis of H1N1 influenza viruses

Eighteen H1N1 influenza virus strains were selected from 42 strains in the library, showing good growth in embryonated chicken eggs (data not shown). The 18 virus strains were antigenically analyzed by hemagglutination-inhibition (HI) test with chicken antisera to H1N1 viruses isolated from birds, pigs and humans (Table 1). The pandemic strain, A/Narita/1/2009 (H1N1) (Narita/09), which was the first isolate in Japan in 2009, reacted with the antiserum to Sw/Hok/81 at a titer of 1:640, 8-fold lower than that to homologous virus. The antiserum to Narita/09 reacted with swine influenza viruses, especially the isolates in 1930–1981 at a titer of 1:1,280-2,560, which was 2- to 4-fold lower than that to homologous virus. These results indicate that the antigenicity of Narita/09 was to some extent related to those of H1N1 classical swine flu virus strains.

**Table 1:** The cross-reactivity of H1N1 viruses isolated from pigs, humans, and birds

	**HI titer of chicken antisera against representative H1 viruses**					
**Virusesa**	**Narita/09**	**Sw/Iowa/15/30**	**Sw/Hok/81**	**PR/8/34**	**Hok/4/96**	**Dk/Mong/540/01**
A/Narita/1/2009	**5,120b**	80	640	40	40	80
**Swine isolates**						
A/swine/Iowa/15/1930	1,280	**1,280**	2,560	20	80	640
A/swine/Niigata/1/1977	1,280	1,280	2,560	40	160	640
A/swine/Shimane/1/1978	2,560	1,280	5,120	40	160	640
A/swine/Shizuoka/1/1978	2,560	1,280	5,120	40	160	640
A/swine/Toyama/1/1978	2,560	1,280	5,120	40	160	640
A/swine/Kanagawa/1/1978	1,280	1,280	640	40	320	640
A/swine/Hokkaido/2/1981	1,280	1,280	**5,120**	80	80	640
A/swine/Miyagi/5/2003 (H1N2)	640	320	2,560	160	80	80
**Human isolates**						
A/PR/8/1934	20	40	40	**2,560**	160	20
A/Hokkaido/2/1996	320	80	80	160	**5,120**	320
A/Hokkaido/11/2002	160	80	80	320	5,120	80
**Avian isolates**						
A/duck/Miyagi/66/1977	160	80	80	40	40	640
A/swan/Hokkaido/55/1996	320	80	40	80	80	1,280
A/duck/Hokkaido/1130/2001	160	80	40	<20	<20	1,280
A/duck/Hokkaido/1203/2001	160	80	80	<20	<20	640
A/duck/Mongolia/540/2001	80	160	40	<20	20	**1,280**
A/duck/Hokkaido/83/2004	160	80	40	<20	<20	640
A/duck/Hokkaido/W73/2007	80	80	80	<20	<20	640

a: Subtype of viruses was H1N1 exept for A/swine/Miyagi/5/2003 (H1N2).

b: Homologous titer was shown in bold.

### Genetic analyses of H1N1 viruses

**Figure 1:**
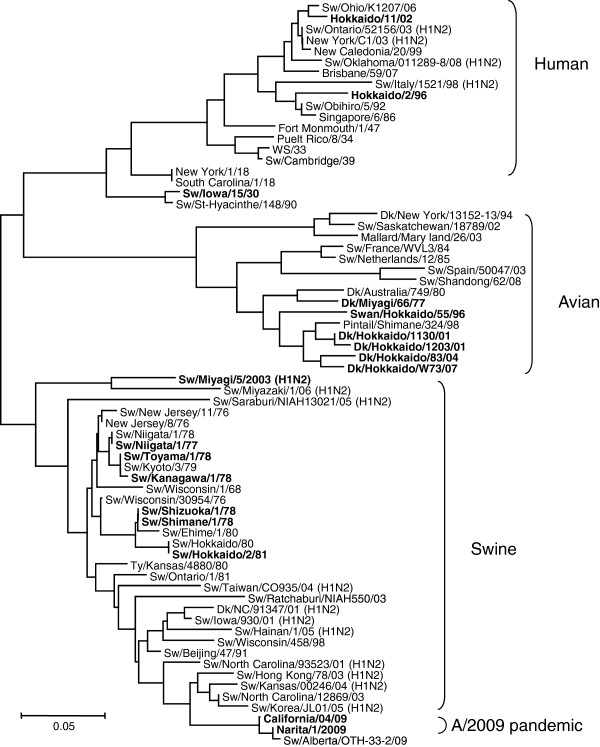
**Phylogenetic tree of H1 HA gene of influenza viruses. **Horizontal distances are proportional to the minimum number of nucleotide differences required to join nodes and sequences. Numbers at the nodes indicate confidence levels in a boot strap analysis with 1,000 replications. Viruses shown in bold were used in this study and classified into 3 groups according to the animal from which they were isolated (human, avian and swine). Subtypes of viruses were H1N1 except for those in parentheses. Sw: swine, Dk: duck.

Nucleotide sequences of the HA genes of the 18 H1N1 viruses were phylogenetically analyzed by the neighbor-joining method with those of other H1N1 strains, including H1N1 viruses isolated from humans. Based on the results of phylogenetic analysis, H1 HA genes were grouped into human, swine, or avian origin clusters (Figure 1). Swine influenza viruses isolated in Japan during 1977–1981 were clustered with pandemic 2009 (H1N1) viruses. Identity of amino acid of HA between Sw/Hok/81 and Narita/09 was 89.9% and glycosylation sites of HA were not different.

### Growth of H1N1 viruses in embryonated chicken eggs

The growth of 18 H1N1 viruses in embryonated chicken eggs was assessed. All the viruses replicated efficiently and had reached a plateau by 48 hours post-infection (p.i.). No significant difference in peak titers of vaccine candidates was detected (data not shown). Sw/Hok/81 showed the highest titer at 10^8.3^ plaque-forming units (PFU)/ml 48 hours p.i., which was 10 times higher than that of Narita/09 (10^7.3^ PFU/ml).

### Potency test of the vaccine against H1N1 pandemic virus in mice

Four, 20, and 100 μg protein of WV or ES vaccines of Narita/09 and Sw/Hok/81, respectively, were subcutaneously injected once into 5 mice. The serum antibody titers of mice against the vaccine and challenge strains were examined (Table 2). The neutralization (NT) antibodies were induced by each vaccine in a dose-dependent manner. Serum NT antibodies induced by injection of WV or ES vaccine of Sw/Hok/81 were not detected with Narita/09.

**Table 2:** Neutralizing antibody titers of mice injected once with the vaccine and virus titers in the lungs after challenge

**Vaccine**			**NT titer to**		**Virus titer in lungs, mean log PFU/g ±Se**^**a**^
**Strain**	**Protein, μg**	**Formulation**	**Narita/09**	**Sw/Hok/81**	
PBS	-	-	<10, <10, <10, <10, <10	<10, <10, <10, <10, <10	5.0±0.17
Narita/09	4	ES	<10, <10, <10, <10, <10	ND	4.6±0.10
	20	ES	<10, 20, 20, 20, 40	ND	4.6±0.12
	100	ES	20, 40, 80, 160, 160	ND	4.1±0.35
Narita/09	4	WV	40, 40, 40, 80, 160	ND	4.2±0.17
	20	WV	40, 80, 160, 160, 320	ND	3.9±0.25*
	100	WV	320, 320, 640, 1280, 1280	ND	2.4±0.50**
Sw/Hok/81	4	ES	<10, <10, <10, <10, <10	<10, <10, <10, 10, 10	4.6±0.04
	20	ES	<10, <10, <10, <10, <10	<10, 10, 10, 20, 80	4.4±0.02
	100	ES	<10, <10, <10, <10, <10	10, 20, 20, 40, 40	4.7±0.02
Sw/Hok/81	4	WV	<10, <10, <10, <10, <10	20, 40, 40, 40, 80	4.5±0.06
	20	WV	<10, <10, <10, <10, <10	20, 40, 80, 80, 80	4.4±0.04
	100	WV	<10, <10, <10, <10, <10	160, 160, 160, 160, 320	4.3±0.09

Mice were injected with each vaccine subcutaneously. Serum samples were collected 3 weeks after injection.

The animals were challenged by intranasal administration of 106.0 PFU of A/Narita/09.

At 3 days after challenge, lungs samples were collected and virus titers were measured. ES: ether split vaccine, WV: whole inactivated vaccine

a: Data are for 5 mice.

*: P<0.05, vs. virus titers in PBS group.

**: P<0.01, vs. virus titers in PBS group.

**Figure 2:**
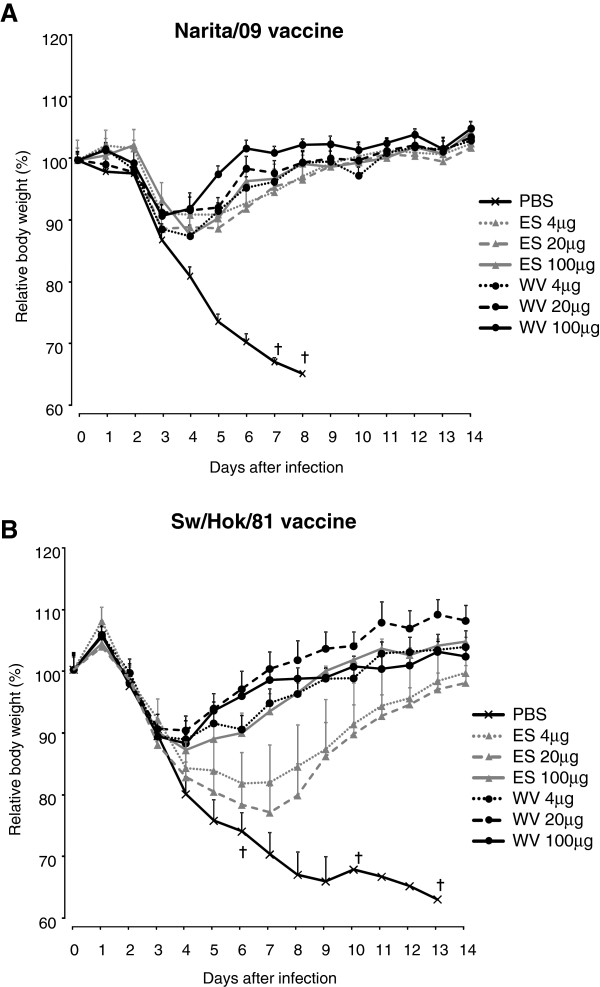
**Changes in body weight of mice injected subcutaneously once with Narita/09 (A) or Sw/Hok/81 (B) vaccine after the challenge with Narita/09. **Data are shown as mean body weight ± standard error. ES: ether split, WV: whole inactivated. †: Mice died.

To assess the potency of the vaccine against the challenge with pandemic 2009 (H1N1) virus, 10^6.0^ PFU of Narita/09 were intranasally inoculated into mice which were injected subcutaneously once with each of the test vaccines. The rate of weight loss of the mice after virus challenge is shown in Figure 2. The mice injected with Narita/09 or Sw/Hok/81 vaccines survived for 14 days, although they showed some weight loss, while the non-vaccinated control mice showed significant weight loss and had died by day 14 after the challenge. In the mice injected with Narita/09 vaccine, no significant difference in weight loss was observed in the mice vaccinated with WV or ES vaccine. The mice injected with ES vaccine of Sw/Hok/81, however, showed significant weight loss compared with mice injected with WV vaccine. The rate of weight loss of mice injected with ES vaccine of Sw/Hok/81 correlated in a dose-dependent manner. The potency of vaccines was also evaluated by measuring the virus titer in the lower respiratory tract of mice (Table 2). The virus titers in the lungs were 10^4.3^–10^4.7^ PFU/g in mice injected with 100, 20, and 4 μg protein of each vaccine of Sw/Hok/81, and 10^5.0^ PFU/g in the non-vaccinated mice.

**Figure 3:**
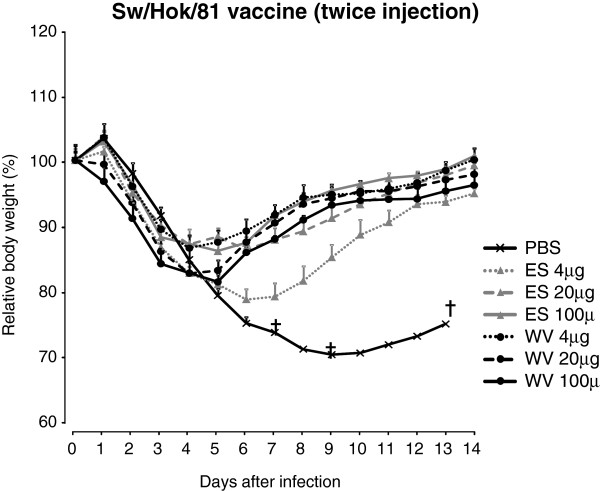
**Changes in body weight of mice injected subcutaneously twice with Sw/Hok/81 vaccine after the challenge with Narita/09. **Data are shown as mean body weight ± standard error. ES: ether split, WV: whole inactivated. †: Mice died.

To improve the efficacy of the Sw/Hok/81 vaccine, WV or ES vaccine of Sw/Hok/81 was injected twice into mice. At 2 weeks p.i., the serum NT antibody titers of the mice injected with the vaccine were higher than that of mice injected once (Table 3). Although the challenge appeared to be less severe compared to first experiment (Figure 3), the virus titers of the lungs of the mice were similar to those of mice injected once with Narita/09 vaccine (Table 3). These results indicate that even if an antigenic difference was observed between vaccine and challenge strains, the WV vaccine induced immunity in mice, decreasing the impact of disease caused by the challenge strain.

**Table 3:** Neutralizing antibody titers of mice injected twice with the vaccine and virus titers in the lungs after challenge

**Vaccine**			**NT titer to**		**Virus titer in lungs, mean log PFU/g ±Se**^**a**^
**Strain**	**Protein, μg**	**Formulation**	**Narita/09**	**Sw/Hok/81**	
PBS	-	-	<10, <10, <10, <10, <10	<10, <10, <10, <10, <10	4.4±0.08
Sw/Hok/81	4.0	ES	<10, <10, <10, <10, <10	20, 40, 80, 160, 160	4.4±0.07
	20	ES	<10, <10, <10, <10, <10	80, 80, 160, 160, 160	4.2±0.19
	100	ES	<10, <10, <10, <10, <10	80, 80, 160, 320, 310	3.9±0.14*
Sw/Hok/81	4.0	WV	<10, <10, <10, <10, <10	160, 320, 320, 640, 640	4.2±0.11
	20	WV	<10, <10, <10, <10, <10	160, 320, 640, 640, 640	3.9±0.28
	100	WV	<10, 10, 40, 40, 160	160, 320, 640, 640, 640	2.9±0.30**

Mice were injected twice with each vaccine subcutaneously with a 2-week interval. Serum samples were collected 2 weeks after the final immunization.

The animals were challenged by intranasal administration of 106.0 PFU of A/Narita/09.

At 3 days after challenge, lungs samples were collected and virus titers were measured. ES: ether split vaccine, WV: whole inactivated vaccine

a: Data are for 5 mice.

*: P<0.05, vs. virus titers in PBS group.

**: P<0.01, vs. virus titers in PBS group.

## Discussion

Vaccination is a measure to reduce the impact of influenza; however, it takes 6 months to prepare a vaccine [16]. Virus isolates from humans usually do not grow well in embryonated chicken eggs, which poses significant limitations for influenza vaccine production. Attempts to increase the yield of candidate vaccine strains have been made by multiple passages in eggs over time or genetic reassortment with a high growth laboratory strain [17,18]. To prepare for pandemic influenza, a virus library of non-pathogenic influenza A viruses with 144 combinations of 16 HA and 9 NA subtypes has been established [15]. In the present study, we selected vaccine strains from 18 H1N1 virus isolates from birds, pigs, and humans on the basis of their growth in embryonated chicken eggs and their antigenicity. Among these viruses, the yield of Sw/Hok/81 in embryonated chicken eggs showed 10^8.3^ PFU/ml, which is higher than that of Narita/09 (10^7.3^ PFU/ml), indicating that a virus strain selected from the influenza virus library could be used for the vaccine strain.

The 1957 and 1968 pandemic influenza virus strains were reassortants of avian and human strains [19]. Kida *et al*. showed that viruses in pigs are in antigenically stasis, as are those in ducks, compared with influenza viruses in humans [9,10]. The present results of antigenic analysis of H1N1 viruses indicate that pandemic 2009 (H1N1) virus was antigenically similar to that of classical swine influenza viruses, not to that of human influenza viruses, as previously described by Garten *et al*. [2]. Although we cannot predict the subtype of the pandemic strain, the antigenicity of the virus is conserved in pigs or ducks. Thus, antigenically related strains isolated from natural hosts could be used for human pandemic influenza vaccines. In order to update the influenza virus library as a seed of vaccine strains, continuous surveillance of avian and swine influenza and the study of pathogenicity, antigenicity, genetic information, and yield in chicken embryo of virus strains are needed.

In the present study, to prepare for future pandemics, we evaluated the potency of a vaccine prepared from Sw/Hok/81 against the pandemic 2009 (H1N1) virus. It was revealed that mice injected with WV or ES vaccine prepared from Sw/Hok/81 induced immunity to suppress the disease manifestation after challenge with Narita/09, although an antigenic difference was observed in these viruses. WV vaccine induces higher immune responses after intramuscular immunization and is superior to ES and subunit vaccine in human populations [20,21]. The reason for these immune responses to WV vaccine is the stimulation of innate [22] and cell-mediated immune responses to internal viral proteins. Indeed, identity of NP protein between Sw/Hok/81 and Narita/09 were 96.9%. In the previous studies, WV vaccine prepared from a virus strain selected from the library also showed protective efficacy against H5 and H7 virus infection in chicken, mice and cynomolgus macaques [23-28]. These results suggest that WV vaccine should work best in immunologically naive people in the early phase of a pandemic and two injections of the vaccine will be more effective even if the antigenicity of the pandemic strain is partially different from the vaccine strain.

## Conclusion

The potency of the vaccine prepared from Sw/Hok/81 for the pandemic 2009 (H1N1) virus was evaluated. Mice injected once with WV vaccine prepared from Sw/Hok/81 induced immunity to suppress weight loss and virus growth in the lungs after challenge with Narita/09. The suppression of virus recovery from lungs of mice injected twice with WV vaccine was similar to that in mice injected once with Narita/09 vaccine. These results suggest that WV vaccine should work best in immunologically naive people in the early phase of a pandemic, and two injections of the vaccine will be more effective if the antigenicity of the pandemic strain is partially different from the vaccine strain.

## Materials and methods

### Viruses and cells

Eighteen H1N1 influenza viruses isolated from humans, pigs and wild birds were used in the present study as representative of 42 H1N1 virus strains in our virus library (http://virusdb.czc.hokudai.ac.jp/). Narita/09 was provided by the National Institute of Infectious Diseases (Tokyo, Japan). Viruses were propagated in 10-day-old embryonated chicken eggs at 35°C for 48 hours.

Madin-Darby canine kidney (MDCK) cells were maintained in minimum essential medium (Nissui, Japan) supplemented with calf serum and used for titration of viral infectivity.

#### Sequencing and phylogenetic analysis

Viral RNA was extracted with TRIzol LS reagent (Invitrogen, Carlsbad, CA, USA) from the allantoic fluid of chicken embryos infected with the virus. Nucleotide sequences of all eight gene segments were determined after RT-PCR, as described previously [29]. The sequence data were analyzed using GENETYX ver. 9.1 (GENETYX Corporation, Tokyo, Japan). Phylogenetic analysis of the HA gene was performed by BioEdit ver. 7.0 and MEGA 5 by the neighbor-joining method with 1,000 bootstraps.

#### Serological tests

HI tests were performed by the microtiter method [30]. The HI titer was expressed as the reciprocal of the highest serum dilution showing complete inhibition of the hemagglutination of 4 HA units of the virus. In NT tests, titers were determined as the reciprocals of serum dilution of the complete inhibition of the cytopathic effect of 100 PFU of viruses using MDCK cells.

#### Viral growth in embryonated chicken eggs

Viruses of 100 50% egg infectious dose (EID_50_) were inoculated into 10-day-old embryonated chicken eggs and incubated at 35°C for 48 hours. Allantoic fluid was harvested to determine viral titers at different time points (0, 12, 24, 48, and 72 hr). The PFU of each virus in the allantoic fluid was determined.

#### Vaccine preparation

To assess the potency of vaccines, inactivated WV vaccines of Sw/Hok/81 and Narita/09 were prepared as described previously [31]. ES vaccine of each strain was also prepared according to Kida *et al.*[32]. Briefly, purified viruses were disrupted with 0.1% Tween 80 and an equal volume of diethyl-ether for 30 min at room temperature. After centrifugation for 30 min at 6,000 g, the water phase was collected and ether dissolved in water was blown out with a stream of nitrogen.

#### Potency test of vaccine against Narita/09 in mice

WV or ES vaccines of each strain with 4, 20 and 100 μg protein were injected subcutaneously into ten 4-week-old female BALB/c mice (CLEA Japan Inc., Tokyo, Japan), respectively. PBS was injected into control mice. Three weeks after immunization, serum samples were collected and 30 µl of 10^6.0^ PFU of Narita/09 was intranasally inoculated into the mice under anesthesia. Three days after the challenge, five mice in each group were sacrificed and the lungs were collected. The virus titers in the lung homogenates were quantified by plaque assay of MDCK cells. Five other mice were observed for clinical signs and weight loss for 14 days. WV and ES vaccines of Sw/Hok/81 were also injected into mice twice with a 2-week interval. Two weeks after the final injection, the serum samples were collected and Narita/09 was inoculated into mice. Animal experiments were authorized by the Institutional Animal Care and Use Committee of the Graduate School of Veterinary Medicine, Hokkaido University (approved numbers: 9148 and 1052) and all experiments were performed according to the guidelines of this committee.

## Competing interests

The authors declare that they have no competing interests.

## Authors’ contributions

MO drafted the manuscript and prepared the vaccines used in the present study. MO, TH, NM carried out animal experiment. YS, and HK participated in the coordination of the study. All authors read and approved the final manuscript.
